# Familial and socio-economic correlates of somatisation disorder

**DOI:** 10.4102/phcfm.v7i1.746

**Published:** 2015-05-11

**Authors:** Abimbola M. Obimakinde, Modupe M. Ladipo, Achiaka E. Irabor

**Affiliations:** 1 Ekiti State University Teaching Hospital, Ado-Ekiti, Nigeria; 2 University College Hospital, Ibadan, Oyo State, Nigeria

## Abstract

**Background:**

Somatisation disorder can result from an interplay between suboptimal family environment and socio-economic deprivation, which enhances the underlying cognitive tendency for this disorder. There are pertinent familial and socio-economic factors associated with this disorder, but research addressing this is sparse.

**Aim and setting:**

The study aims to evaluate family and socio-economic factors that are associated with somatisation disorder amongst patients presenting to the Family Medicine clinic, University College Hospital, Ibadan, Nigeria.

**Methods:**

This is an observational case-control study of 120 participants who presented to the clinic between May and August 2009. Data collection was by interviewer-administered structured questionnaire using the World Health Organization Screener for Somatoform Disorder and Somatoform Disorder Schedule to ascertain somatisation in 60 patients who were then matched with 60 controls. The respondents’ demographic and family data were also collected and their interpersonal relationships were assessed with the Family Relationship Index.

**Results:**

The somatising patients were mostly females (70%), with a female to male ratio of 2.3:1 and mean age of 43.65 ± 13.04years.Living in a polygamous family (as any member of the family) was significantly related to somatisation (*p* = 0.04). Somatisation was also more common in people who were separated, divorced or widowed (*p* = 0.039). Somatisers from a lower social class or those earning below a dollar a day experienced poorer cohesion (*p* = 0.042) and more conflicts (*p* = 0.019) in their interpersonal relationship.

**Conclusion:**

This study was able to demonstrate that a polygamous family setting, disrupted marriage, low social status and financial constraints are correlates of somatisation. It is of essence to identify these factors in holistic management of somatising patients.

## Abstrait

**Corrélations familiales et socio-économiques des troubles de somatisation.**

**Contexte:**

Les troubles de somatisation proviennent d'une interaction entre l'environnement familial sous-optimal et le dénuement socio-économique qui augmente la tendance cognitive sous-jacente à ces troubles. Il y a des facteurs familiaux et socioéconomiques pertinents associés à ces troubles, mais il y a peu de recherches sur ce problème.

**Objectif et cadre:**

L’étude a pour but d’évaluer les facteurs familiaux et socio-économiques qui sont associés avec les troubles de somatisation parmi les patients de la clinique de médecine familiale de l'University College Hospital, Ibadan, Nigeria.

**Méthodes:**

Ceci est une étude cas-témoin de 120 participants qui se sont présentés à la clinique de mai à août 2009. La collecte de données a été faite au moyen d'un questionnaire structuré géré par l'interviewer qui a utilisé le questionnaire de l'Organisation mondiale de la Santé pour les troubles somatoformes et le programme des troubles somatoformes pour vérifier la somatisation chez 60 patients qui ont été appariés à 60 contrôles. Les données familiale et démographiques des participants ont aussi été collectées et leurs relations interpersonnelles ont été évaluées au moyen de l'Indice des Relations familiales.

**Résultats:**

Les patients somatisant étaient principalement des femmes (70%), avec un rapport homme-femme de 2.3:1 et un âge moyen de 43.65 ± 13.04 ans. Le fait de vivre dans une famille polygame (en tant que membre de la famille) était fortement lié à la somatisation (*p* = 0.04). La somatisation était aussi plus courante chez les personnes séparées, divorcées ou veuves (*p* = 0.039). Les personnes d'origine modeste ou gagnant moins d'un dollar par jour souffrant de somatisation avaient moins de cohésion (*p* = 0.042) et plus de conflits (*p* = 0.019) dans leurs relations interpersonnelles.

**Conclusion:**

Cette étude a pu démontrer qu'une situation de famille polygame, un mariage perturbé, un statut social défavorisé et des contraintes financières sont en corrélation avec la somatisation. Il est essentiel d'identifier ces facteurs pour la gestion holistiques des patients somatisant.

## Introduction

Somatisation disorder is a chronic condition which is characterised by arrays of vague physical complaints recurring over years.^1^ The disorder is highly stigmatised and there is positive evidence with a strong presumption that the symptoms are linked to psychosocial factors.^1,2^ In the medical community little is known about the epidemiology of somatisation disorder, despite the enormous burden of the disease on patients and the healthcare system.^3^

It has been estimated that about one-third of all patients presenting with functional complaints to primary care providers have associated socio-economic and family problems.^1,2^ Somatisation is seen to be a somatic manifestation of psychological discomfort.^2,3^ Humans are not isolated entities but the result of continuous interaction with each other and situations.^4^ It has been said that ‘Individuals cannot be understood in isolation from one another, but as a part of their family, as the family is an emotional unit’.^5^ Family members may not accompany a patient into the consulting room; nevertheless, the family's influence on a patient's health is always palpable.^4,6^ An individual usually exists within a family, which is the natural and basic unit of a society and the family plays an important role in influencing the behavioural pattern of patients.^5^

Somatisation disorder is characterised by physical symptoms that mimic disease or injury, for which there is no identifiable physical cause.^1,2,7^ The physical complaints also cannot be explained in terms of the results of substance abuse, or by other mental disorders.^8^ The medical test results in these patients are either normal, or do not explain their symptoms, or the exhibited symptoms are inappropriately in excess of an identifiable cause.^2,7^

The physical symptoms may reflect a plea for help and a desire to be cared for. There is often a long history of unexplained physical symptoms and frequent use of healthcare centres. Patients typically deny or minimise their emotional distress and their associated life adversity.^1,7^ Somatisation disorders may have developed as a result of an interaction between personal vulnerability and negative life events, which in turn may have activated the underlying cognitive predisposition.^3,6,9^ Family members may also model somatising behaviour for their children or each other.^3^

The pattern of interaction that emerges in a family system helps to maintain the family's equilibrium and provide clues to each member about how they should function.^5^ Irrespective of the boundary of a family, the influence of family on an individual's functioning is shaped more by the family dynamics and its environment.^5^ Within the confines of the family system patterns of interaction develop, as certain family members’ behaviour affects other family members’ behaviours in predictable ways.^5^ Family member interactions with each other often have a strong influence on the way people see themselves and the world, and influence their relationships, behaviours and wellbeing.^6,9^ Symptomatic behaviours are seen as arising out of the interrelated behaviour of all family members; therefore, in order to gain a better understanding of a person's situation, their behaviour is explored in the context of their family system.

Some of the many influences on family dynamics, especially early in life, include the nature of the parents’ relationship, marital status, finances, the ‘mixture’ of members who are living in the same household, extended family, values, culture, and events which have occurred and shaped them. All or any these influences are possible deterrents or inciters of somatisation. Adults with somatisation are commonly from a lower socio-economic class with less education and poor vocational skills or exist within a chaotic family setting.^1,2^ This may not be a far-fetched scenario in the polygamous family with its attendant adverse socio-economic issues, especially in the typical West Africa family setting.

Pertaining to the lower socio-economic class, it may be less acceptable to express one's emotion directly, and therefore emotions are preferably somatised.^6,9,10^ Inadvertently, due to the peculiar hardships and stressful negative life events experienced in the lower social class, raising emotional issues may not be tolerated or is considered a trivial matter.^6,9,10^ Single parenthood, a dysfunctional family setting, living alone with a predisposition to lack of an avenue for expression of feelings, and unemployment are amongst other predisposing factors to somatisation.^1,11^ Other interpersonal traumas that have been implicated in this disorder include severe marital difficulties, separation, neglect, emotional abuse, witnessing violence, and physical and sexual abuse.^1,2,3^

Freud's early theories brought to light the fact that if the individual could maintain his psychic equilibrium by himself, apparently he would have little need for other people.^2,3^ Admittedly this theory acknowledges the importance of some familial interaction, society and the role of culture and relationships in the ego building of an individual.^1,3^ The assessment of family dynamics is not to judge a family as healthy or unhealthy, but to help a clinician understand why the individual in the context of his family has an illness, and which factors in the family system, if any, are contributing to, affected by, or complicating the illness.^5^

In a home environment characterised by conflict either between the parent and child or both parents, the child and/or the parent may begin to prefer to internalise feelings and express somatic problems more frequently.^6,9^ A sensitive or emotionally reactive person who perceives more threats and dangers of any form in the family environment, be they real or imagined, may more likely use somatic complaints in signalling others to help cope with distress.^6^ It is interesting to note how each person chooses to cope with their psychological turmoil: some share their emotional burden with significant others, some write in their diaries, whilst some keep issues bottled up within, suffering greater distress as traumatising life experience evolves.^1,3^ This final group of individuals represents the largest group of somatisers, most often presenting with the most severe symptoms. Of all dimensions of family dynamics, family conflict resolution, level of family cohesion and expressiveness of emotions are the important predictors of somatisation.^12,13^

In essence it has been concluded that somatising patients are from families that are less supportive, less cohesive, and less adaptive than other families.^12,13^ This behaviour might be maintained by family conflict, and this effect might be stronger in socially disadvantaged homes with financial and social constraints, which could potentially increase stress and conflict in the home.^6,12,13^ Somatisation often presents puzzling problems for the family physician to overcome. One should acknowledge that the aetiology of somatisation involves a combination of factors. These patients often have a history of inadequate coping, painful interpersonal relationships and frequent unsatisfying relationships with healthcare providers. Despite all these known facts there is a paucity of literature addressing these associations.

It is pertinent for family physicians to heed the life issues that may be the distressing factors in these patients, instead of viewing them as difficult patients and attempting to dismiss them quickly, as this attitude generally worsens their symptoms. Exploration and treatment of the biopsychosocial context may hold the key to solving the problems in somatising patients.

## Research methods and design

### Setting

The study was carried out at the Family Medicine outpatient clinic of the University College Hospital (UCH), Ibadan. Ibadan is the capital of Oyo State, situated in the southwestern region of Nigeria. Virtually all of Nigeria's ethnic groups are represented here, with a preponderance of the indigenous people of Yoruba ethnicity. The indigenous people are socially and culturally conscious, with a strong sense of family ties. The clinic is the entry-point for most patients presenting to the UCH in Ibadan, where they are attended to by consultant and resident family physicians with referral to other specialties when appropriate.

### Study population and sampling strategy

There were 120 participants enrolled for the study, comprising 60 adult patients with somatisation disorder and 60 adult patients in the control group. These were patients presenting to the Family Medicine clinic of the UCH between May and August 2009.The sample size was an estimate using the formula for comparative study [*n* = (2z^2^pq)/*d*^2^] incorporating the prevalence of somatisation from a previous local study.^11^ The calculated sample size has a statistical power of 0.80 using Power Analysis and Sample Size Software version 13(PASS 13).

A total of 2668 adults presented to the outpatient unit during the study period, and all who consented were screened for somatisation using a validated structured questionnaire administered by the researchers, attending physicians and research assistants. Consecutive individuals who satisfied the screening criteria were then administered the diagnostic tool. Respondents who satisfied the initial screening but did not fulfil the diagnostic criteria were dropped from the study during the selection process. There was eventual identification of 60 eligible respondents who satisfied both screening and the diagnostic criteria for somatisation.

The selected somatising patients were then matched with a control group using age (with difference of ± 2 years), sex and level of education. The control group comprised those who consented to participate in the study and who were also verified not to be somatisers by administration of both the screening and diagnostic criteria. Non-consenting patients and patients with other diagnosed mental health issues were excluded from the study.

### Data collection

The survey was administered using standardised interviewer-administered questionnaires. The World Health Organization (WHO) Screener for Somatoform Disorders (SSD) was used as screening tool for somatisation. This is a 12-item questionnaire developed by experts to identify patients likely to present with somatoform disorder.^14^ A positive response to at least 3 of the 12 screening questions in the previous 3 months qualified the patient for recruitment into the study. The disorder was further verified using the WHO Somatoform Disorder Schedule (SDS),^14^ which includes 14 items that strictly assess for somatisation. A positive response to at least 6 of the 14 symptoms spanning at least two years is diagnostic of somatisation. Both tools are validated instruments with high inter-rater reliability and test-retest diagnostic reliability.

Information on demographic characteristics of the respondents such as age, gender, and highest educational qualification, occupation, approximate monthly wage, religion, and ethnic group was obtained. Occupation was later used for classification into social class according to the occupational grouping of Boroffka and Olatawura.^15^ Information on type of family of origin, marital status, and the type of present family setting were also obtained.

The Family Relationship Index (FRI) is a 27-item self-report measure that provides an overall index of the quality of the family dynamics, as assessed by the family's degree of cohesion, expressiveness, and conflict resolution.^16^ Participants were asked questions related to these three dimensions of family dynamics. Nine items are used to assess each: cohesion, expression and conflict. The response format to the questions of the FRI is a two point one (true or false), and total response is summed individually as a score of 9 each for the three dimensions.

### Data analysis

Frequency tables were generated for relevant variables. Descriptive statistics such as mean and standard deviations were used to summarise quantitative variables, whilst categorical variables were summarised with proportions and percentages. The Chi-square test was used to investigate associations between categorical variables, whilst the independent sample *t*-test was used to test for differences between two mean values. Analysis of variance (ANOVA) was used to test for between-subject effects. Level of statistical significance taken as *p* < 0.05. The data were analysed with Statistical Package for the Social Sciences (SPSS) software version 16 after sorting and coding the questionnaire

### Ethical considerations

Ethical clearance for the study was obtained from the joint University of Ibadan/UCH ethical review board. Informed consent was sought and obtained from each study subject recruited, in accordance with ethical principles for the guidance of physicians in medical research.

## Results

The mean age of the somatisers studied was 43.65 ± 13.04 years, which is similar to that of the control group (mean age 43.95 ± 13.37 years) (*p* = 0.77). Details of the demographics are as shown in Table 1.

**TABLE 1:** Demographic characteristics of the somatisers and the control group.

Variable	Somatisers *n = 60*		Control *n = 60*		Total *n = 120*		χ2	*p*-value
	*n*	%	*n*	%	*n*	%		
**Age (yrs)**								
≤19	0	0	1	1.7	1	0.8		
20–39	24	40.0	24	40.0	48	40.0		
40–59	25	41.7	23	38.3	48	40.0		
60–79	11	18.3	12	20.0	23	19.2	1.127	0.771
**Marital status**								
Married	36	60.0	37	61.7	73	60.8		
Separated/divorced	4	6.7	8	13.3	12	10.0		
Widowed	8	13.3	4	6.7	12	10.0		
Single	12	20.0	11	18.3	23	19.2	2.724	0.435
**Religion**								
Christianity	34	56.7	37	61.7	71	59.2		
Islam	25	41.7	23	38.3	48	40.0		
Traditional	1	1.7	0	0.0	1	0.8	1.210	0.0546
**Ethnicity**								
Yoruba	47	78.3	49	81.7	96	80.0		
Igbo	6	10.0	4	6.7	10	8.3		
Hausa	3	5.0	5	5	8	6.7		
Others	4	6.7	2	3.3	6	5.0	1.608	0.658
**Occupational class**								
Class I–II	14	23.3	18	30.0	32	26.7		
Class III–IV	17	28.3	22	36.7	39	32.5		
Class V–VI	29	48.3	20	33.3	49	40.8	2.794	0.247

### Family setting

**FIGURE 1:**
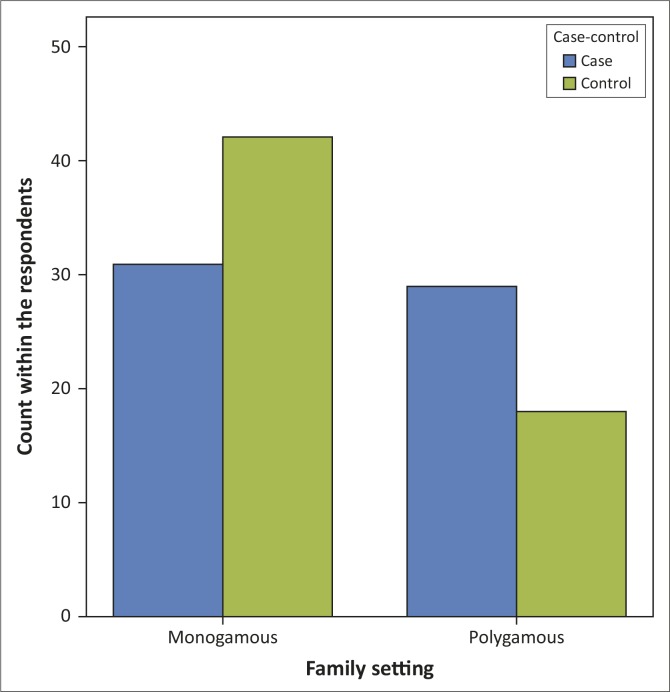
Respondents’ family setting.

Approximately 50% of the individuals with somatisation were currently in polygamous family settings, with 30% of the control group living in a polygamous family setting, at a statistical significance of *p* = 0.04, as shown in Figure 1.

### Family of origin

Most of the respondents were originally from polygamous families, but there was a higher percentage amongst the somatisers in comparison to the control group. Further analysis of the impact of family of origin on family dynamics of the respondents revealed results as shown in Table 2. Somatising patients from polygamous origin have higher scores (mean 3.42) on conflict with a *p*-value of 0.054, which is not significant but is a noticeable difference.

**TABLE 2:** Comparison of family dynamics score by family of origin.

Variable	Somatisers, *9*n = 9 Controls		
	Mean score (SD)	Mean score (SD)	*P*-values
**Cohesion**			
Monogamous	6.47 (3.93)	8.05 (2.21	
Polygamous	6.40 (3.45)	7.84 (2.64)	
**Total Score**	**6.42 (3.56)**	**7.92 (2.47)**	**0.804**
**Conflict**			
Monogamous	2.41 (3.22)	0.64 (1.26)	
Polygamous	3.42 (2.81)	1.26 (1.90)	
**Total Score**	**3.13 (2.94)**	**1.03 (1.71)**	**0.054†**
**Expression**			
Monogamous	6.71 (2.95)	7.82 (1.59)	
Polygamous	5.60 (2.78)	7.11 (2.50)	
**Total Score**	**5.92(2.85)**	**7.37(2.23)**	**0.083†**

†, There is a noticeable impact of family of origin on conflict and emotional expression between the two groups.

*n* = sum of each dimension of the family relationship index.

### Family dynamics score by marital status

Analysis of marital status in relation to its effect on family dynamics revealed that the married somatisers had lower scores on cohesion (mean 6.78) in comparison to married controls (mean 8.32); amongst the somatising patients the respondents not currently living with their spouses (absent spouse) had the lowest scores (mean 5.58, df = 2, *p* = 0.039), which is statistically significant. The somatisers, irrespective of their marital status, had higher scores (mean 3.13) for conflict and lower scores for expression, as shown in Table 3.

**TABLE 3:** Mean scores of family relationship index considering marital status.

Variable	Somatisers, *n* = 9 Controls		
	Mean score (SD)	Mean score (SD)	*P*-values
**Cohesion**			
Married	6.78 (3.38)	8.32 (2.07)	
Single	6.17 (3.56)	8.73 (0.65)	
Absent Spouse	5.58 (4.20)	5.93 (3.58)	
**Total**	**6.42 (3.56)**	**7.92 (2.47)**	**0.039**
**Conflict**			
Married	3.03 (2.84)	0.86 (1.51)	
Single	3.17(3.16)	1.00 (1.27)	
†Absent Spouse	3.42 (3.26)	1.58 (2.50)	
**Total**	**3.13 (2.94)**	**1.03 (1.70)**	**0.629**
**Expression**			
Married	6.08 (2.80)	7.70 (1.84)	
Single	5.17 (3.27)	7.64 (1.96)	
Absent Spouse	6.17 (2.65)	6.08 (3.12)	
**Total**	**5.92 (2.85)**	**7.37 (2.22)**	**0.388**

†, Absent spouse connotes separated, divorced or widowed respondents.

*n* = sum of each dimension of the family relationship index.

### Comparison of socio-economic variables of somatisers with controls

**FIGURE 2:**
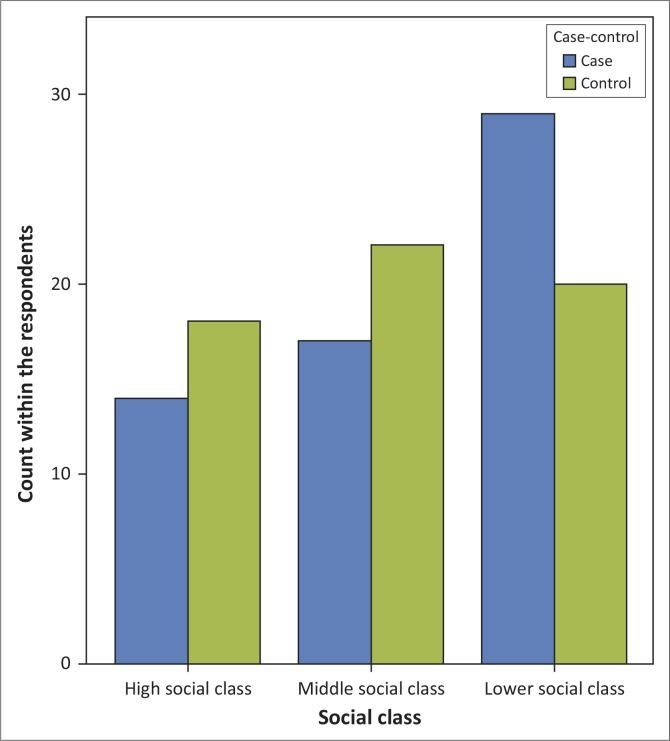
The social class of cases and controls.

Somatising patients were more likely to be of lower socio-economic status; however, despite this observed trend there was no significant difference between the somatisers and the control group (*p* = 0.247), as depicted in Figure 2.

The somatisers had lower scores (mean 6.42) for cohesion in comparison to the control group (mean 7.92, *p* = 0.379) across the social classes, but within the somatising group individuals from middle and low social class had lower scores. The control group had more scores for expression (*p* = 0.079), but within the somatisers those from the middle (mean 5.71) and lower class (mean 5.72) had the least scores whilst those in the high social class had more scores (mean 6.57). Conflict scores were higher for the somatisers in comparison to the non-somatisers, but within the somatising group those from a high social class had the least scores on conflict. None of these observed differences were statistically significant, as shown in Table 4.

**TABLE 4:** Impact of social class on family dynamics score.

Variable	Somatisers, *n* = 9 Controls		
	Mean score (SD)	Mean score (SD)	*P*-values
**Cohesion**			
High social class	7.00 (3.04)	7.56 (2.69)	
Middle social class	5.59 (4.09)	7.68 (2.92)	
Lower social class	6.62 (3.49)	8.50 (1.61)	
**Total**	**6.42 (3.56)**	**7.92 (2.47)**	**0.379**
**Conflict**			
High social class	2.76 (2.77)	1.71 (1.79)	
Middle social class	3.76 (3.36)	1.18 (2.04)	
Lower social class	3.14 (2.82)	0.75 (1.21)	
**Total**	**3.13 (2.94)**	**1.03 (1.71)**	**0.392**
**Expression**			
High social class	6.57 (2.14)	7.28 (2.17)	
Middle social class	5.71 (3.26)	7.32 (2.49)	
Lower social class	5.72 (2.94)	7.50 (2.07)	
**Total**	**5.92 (2.85)**	**7.37 (2.22)**	**0.079**

*n* = sum of each dimension of the family relationship index.

**FIGURE 3:**
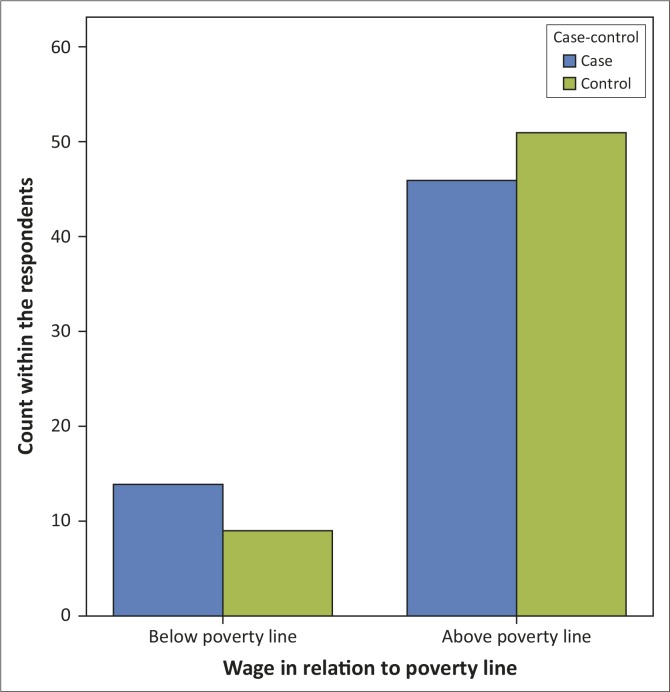
Distribution of respondents by wage category.

Only 23 of the 120 participants in this study were earning or living on less than a dollar a day, which is the WHO cut-off for poverty. Of these, 14 (60.9%) were in the somatising group (Figure 3). Further analysis revealed that the somatising group earning below the poverty line had lower scores for cohesion (mean 7.36, *p* = 0.042) and expression (mean 5.50, *p* = 0.67) in comparison to the control group in this category. In contrast, the control group had mean scores of 8.56 for cohesion and 7.67 for expression. Following the same pattern, the somatisers earning below the poverty line had more scores for conflict (mean 2.21) compared to the controls (mean score 0.11) (*p* = 0.019) (Table 5).

**TABLE 5:** Comparison of impact of poverty by wage classification on family dynamics.

Variable	Somatisers, *n* = 9 Controls		
	Mean score (SD)	Mean score (SD)	*P*-values
**Cohesion**			
Below poverty line	7.36 (2.62)	8.56 (0.88)	
Above poverty line	6.31 (3.78)	7.80 (2.65)	
**Total**	**6.42 (3.56)**	**7.92 (2.47)**	**0.042†**
**Conflict**			
Below poverty line	2.21 (2.42)	0.11 (0.33)	
Above poverty line	3.41 (3.05)	1.20 (1.80)	
**Total**	**3.13 (2.94)**	**1.03 (1.70)**	**0.019†**
**Expression**			
Below poverty line	5.50 (2.87)	7.67 (1.73)	
Above poverty line	6.04 (2.86)	7.31 (2.31)	
**Total**	**5.92 (2.85)**	**7.37 (2.22)**	**0.675**

†, There is a statistically significant difference for effect of earned wage on levels of cohesion and conflict between the two groups.

## Discussion

The somatising patients in this study were in the age range of 21–78 years, and the majority (70%) were married females from a lower social class, which corroborates with the current literature.^2,3^

### Family structure and somatisation

The somatising patients in this study, in contrast to the controls, were mostly born and raised (71.7%) or currently living (50%) in a polygamous family setting (Figure 1). The practice of polygamy is widespread in many areas of the world, and varies from culture to culture. This is the case amongst the Yorubas, even when Christianity is the religion practised, as observed in the groups studied. Polygamy can have deleterious effects on the health of either gender and at any age, as the practice is associated with stress, tension and disequilibrium in the family structure.^17,18^

It seems the females in the polygamous family suffer the most psychological distress, and it is important to note that the majority of somatisers are women, as exemplified in this study, where 70% of the participants were females. It can be assumed that these somatising females, who were mostly married, suffer emotional distress as partners of polygamous men. Previous studies examining the psychosocial profile of some women living in polygamous and monogamous marriages found that women in polygamous marriages reported lower levels of marital satisfaction, paranoid ideation and higher levels of somatisation, which is perhaps the situation amongst the women studied.^17,18^

The high levels of rejection and hostility amongst fathers and mothers, especially in polygamous families, are strongly correlated with and predictive of somatisation in any member of the family.^17^ This is coupled to the fact that physical and/or psychological *violence –* which may occur in polygamy and even monogamous relationships – has been known to heighten rates of internalising symptoms, which is related to somatisation.^18^ Inklings of psychological violence can be inferred from the results seen in Table II, where the FRI scores for those raised in polygamous setting revealed a higher mean conflict score (3.42) for the somatisers (*p* = 0.054), with lower scores for cohesion and expression of emotion. The higher conflict rating for those from polygamous families, as seen in this study, maybe a reflection of the assumption that affection has to be shared or earned, as has been observed in the traditional West African polygamous setting.

There is a greater prevalence of various somatic symptoms, low self-esteem and loneliness amongst individuals from or in polygamous settings.^19^ The senior wives have been known to perceive more dissatisfaction and rejection and thus report more psychological distress.^17,18,19^ However, it is also noted that polygamous marriages can have a very deleterious effect on the mental health of second wives or any other of the wives in such families.^20,21^

Although this study revealed female preponderance, to come to the aforementioned conclusion it could have been more beneficial to relate their position as a wife to their husbands, but this information was not sourced. Ebigbo et al.^22^ also reported that in polygamy competition between the wives, overburdening of the husband and often poor care of the children represent the background for development of psychosomatic symptoms in any individual living in this type of family setting. Any individuals in polygamous families can show significant levels of psychological distress, and more problems in family functioning and life satisfaction.^18,20,21^ This is exemplified in this study, where although the somatisers were mostly females, a sizable 30% were males, stressing the need also to accord men biopsychosocial evaluation in management of this disorder, as men could also suffer significant psychological distress as a consequence of unhealthy family dynamics.

### Marital status and somatisation

The somatisers who were not currently living with their spouses had the lowest scores on family cohesion (5.58 out of 9) and higher conflict scores; this is in keeping with studies that pointed out that somatisers are less likely to be married or living with a partner, as the disorder is associated with being separated, widowed ordivorced.^11,23^ The reason for this association could be explained by the fact that those divorced or separated individuals are in their present situation due to unresolved conflict, poor cohesion and inefficient emotional expression.

Evidence suggests that open communication in a marriage may facilitate psychological well-being.^24,25^ Avoiding problems may ultimately be more destructive than trying to work out relationship issues.^6,25^ The concept of ‘short-term pain, long-term gain’ has been reported in the marital relationship literature, where disagreement and anger exchange between couples was associated with an increase in marital satisfaction and good psychological wellbeing.^24,25^ However, effective communication seemed to be lacking in the somatisers in this study, as their overall expression score (5.92) was much lower than that of the controls (7.37) (*p* = 0.388),which might have contributed to the disorder.

Doohan et al.^25^ reported that poorly managed marital conflict is associated with a range of behavioural and emotional problems, which may manifest as somatisation. This is reflected in this study, where analysis based on marital status showed that somatisers’ overall cohesion scores is lower (6.42) in comparison to the controls (7.92), with statistical significance of 0.039. Also with respect to the patients’ marital status the somatisers had higher conflict score (3.13) than the controls at *p* = 0.629, but this was not a significant finding. The family dynamics scores by marital status can be attributed to emotional issues within the marriage union. These individuals could have been exposed to a certain degree of family instability and disruption, with resultant psychological distress and a tendency to somatisation.

### Socio-economic status and somatisation

The social classification was done based on respondents’ occupation, into high, middle and low social class. The result (Figure 2) showed that more (67%) of the controls were from the high and middle social strata, and about half (48.8%) of the somatisers belonged to the lower social class, despite the fact that the two groups were matched by educational attainment. It was again evident that somatisation is more prevalent amongst individuals in the lower social strata, although the result was not statistically significant.^11,23^ This shows that those somatisers belonging to a lower social class but with a higher educational attainment maybe experiencing some sort of psychological distress due to dissatisfaction with their social status and placement within society.

Its known that belonging to a higher social class and working full-time appears to be protective against somatisation, as it increases the individual's sense of psychological wellbeing.^24^ This could be a consequence of the fact that individuals from higher social classes inadvertently would be more enlightened and as such would then be more rational in diffusing emotional turmoil, and psychologise in preference to somatisation.^3,24^

Table IV showing the family dynamics scores of the different social classes revealed that somatisers belonging to the middle and lower social class had lower scores on cohesion (5.59, 6.62) and expression (5.71, 5.72), with associated higher conflict scores (3.76, 3.14), although these results are not statistically significant. In contrast, the controls had better scores for FRI, which buttresses the fact that the financially buoyant high social class family which can afford outing times together is provided an avenue that promotes cohesiveness and the opportunity to work at conflict resolution, unlike in the lower social class where trying to overcome the hassles of life may leave no room for such.^2,3,26^

Categorisation of the income of the respondent revealed that more of the somatising patients (23.3%), in comparison to 15% of the controls, were living below the poverty line based on their monthly wage, as seen in Figure 3. Poverty has been found to be an important factor in part-time workers, the unemployed and other economically inactive individuals (mostly retired or housewives), and it strongly predicts somatisation.^11,26^ There is an association between poor mental health and low income level or social deprivation.^27^

Also, the literature indicates the existence of links between poverty and a high level ofneuroticism.^27^ It has been identified that a modest tendency for somatisation is more common in individuals with lower education, lower income, poverty and joblessness.^26,27^ In addition, as shown in Table V, the somatisers living below the poverty line (in contrast to similar controls) also had lower scores on cohesion (mean = 7.36, *p* = 0.042), with higher scores on conflict (mean = 2.21, *p* = 0.019).This result is supported by other reports on the tendency for the life situation of individuals in lower socio-economic strata to be affected by chronic stress of financial incapability and predisposition to more conflict within the family.^27^

Poverty and joblessness intensified issues within the family, and there are also weak family ties in the lower social class.^26^ Social deprivation amongst those living below the poverty line or at the lower social strata magnifies the effects of other stressors in the family and renders the individuals particularly vulnerable. The problematic structure of the socio-economically disadvantaged has been reported as the root cause of somatisation symptoms in individual family members.^6,12,23^ A socio-economically stable family faced with a disruptive stressor can adapt in response to the changing needs of family members, thus protecting vulnerable family members from experiencing negative psychological sequelae.^26,27^ It was obvious from this study that the dynamics at work in individuals’ family lives as consequences of their socio-economic status had some bearing on the tendency for somatisation disorder.

### Recommendation

In view of the findings of this study, it will be beneficial routinely to consider and properly evaluate the family history and socio-economic details when attending to somatising patients. Incorporating this extra effort, especially in the context of their interpersonal relationships, may reduce the burden of somatisation on both the patients and the attending physicians.

## Conclusion

It can be inferred from this study that living within a polygamous setting or marriage, earning or living below the poverty line, being of lower social class and being widowed, separated or divorced are significant familial and socio-economic correlates of somatisation disorder. The family and social environment of these somatisers revealed lack of emotional closeness and poor cohesion and conflict resolution, which could have enhanced the tendency for somatisation.
